# Multilevel subdural and epidural hematoma after percutaneous kyphoplasty (PKP): a case report and literature review

**DOI:** 10.3389/fmed.2024.1499630

**Published:** 2025-01-20

**Authors:** Fuyuan Deng, Hailong Liu

**Affiliations:** Department of Orthopedics, Deyang People’s Hospital / Orthopedic Center of Deyang City, Deyang, China

**Keywords:** spinal epidural hematoma, spinal subdural hematoma, percutaneous kyphoplasty, hypertension, spinal cord compression

## Abstract

**Background:**

Serious complications such as intraspinal hematoma after percutaneous kyphoplasty (PKP) are rare, with an incidence of about 1%, which has been reported in relevant literature. However, there are few reports on the simultaneous occurrence of multiple segmental subdural/epidural hematomas (SDH/SEH) after surgery.

**Case presentation:**

This case reports a 90-year-old female with lower limb neurological dysfunction after PKP. Emergency thoracolumbar magnetic resonance imaging (MRI) showed hematoma compressing the spinal cord. After conservative treatment, the sensory and motor function of the right lower limb returned to normal, and the bowel and urine function returned to normal, but the muscle strength of the left lower limb did not fully recover.

**Conclusion:**

Blood pressure should be well controlled during the perioperative period for elderly patients with hypertension. This will avoid severe fluctuations in blood pressure caused by pain, anesthesia, and other reasons, reduce the probability of intraspinal vascular rupture, and prevent severe neurological dysfunction caused by acute intraspinal hemorrhage.

## Background

PKP is an effective method for the treatment of osteoporotic fractures and some vertebral bone tumors, and most patients have good curative effects. Because it is less invasive, the operation can be completed under local anesthesia, and has little effect on the systemic function of patients, it has been widely used in clinical practice (1–3). However, PKP has some serious complications, such as spinal cord or nerve root injury, subdural or epidural hematoma, infection, pulmonary embolism, and so on (2, 4–6).

Acute Spinal epidural hematoma (7) and subdural hematoma (1) are rare complications after PKP, which usually occur within a few hours after surgery, and patients will experience severe neurological dysfunction in a very short time. According to current literature reports, open decompression surgery should be considered in time for patients with spinal cord compression (7, 8). We present a case of multisegmental subdural and epidural hematoma after PKP. We excluded the common causes of spinal cord compression in patients after PKP was reported in the literature. We considered that it was caused by intraspinal vein rupture and bleeding caused by intraoperative blood pressure fluctuation. After relevant treatment, the patient’s neurological dysfunction was recovered.

## Case presentation

**Figure 1:**
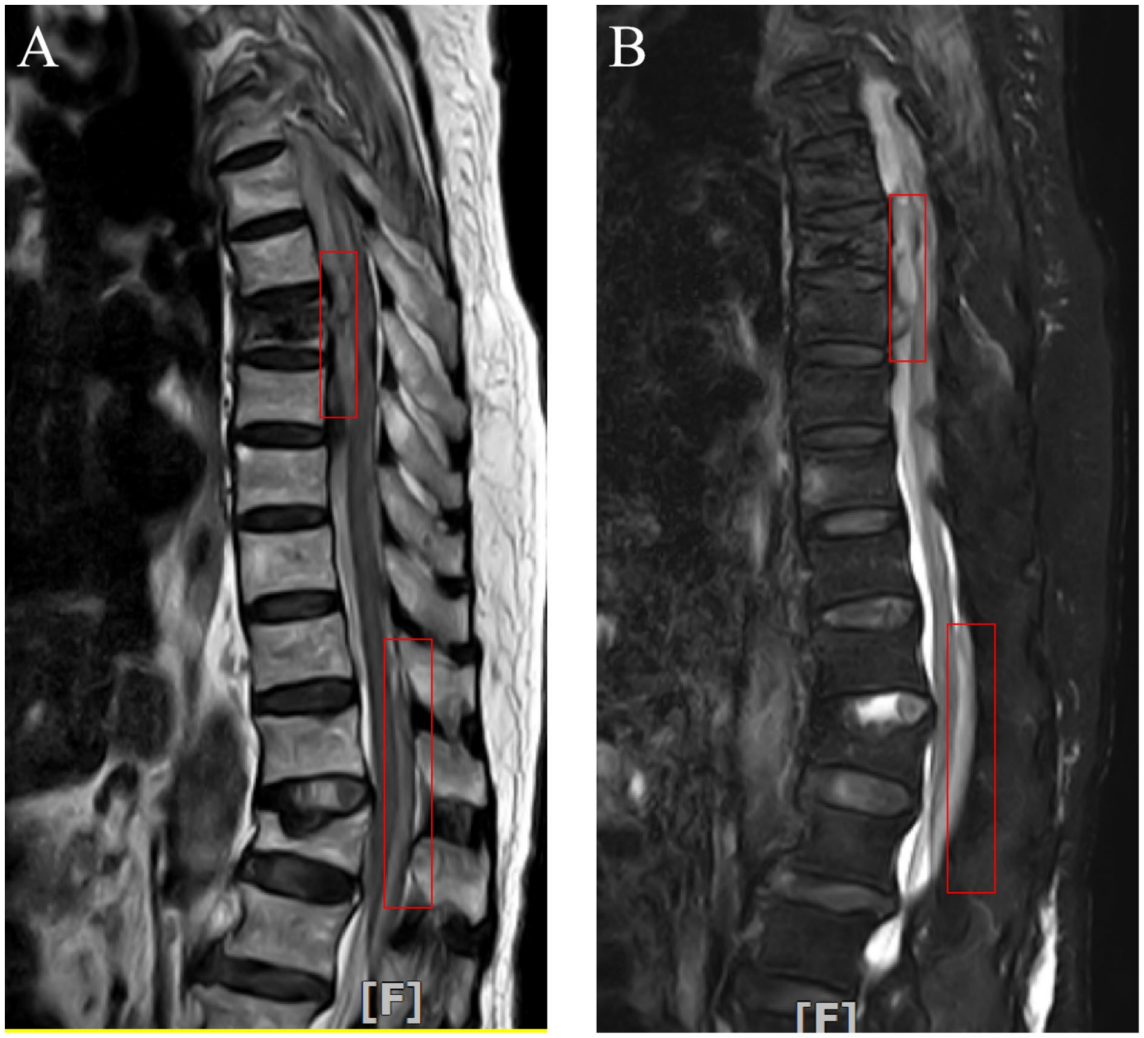
Postoperative thoracic MRI revealed subdural hematoma in front of the spinal cord at the T4-8 vertebral plane and epidural hematoma behind the spinal cord at the T12-L2 vertebral plane (Red box marked).

**Figure 2:**
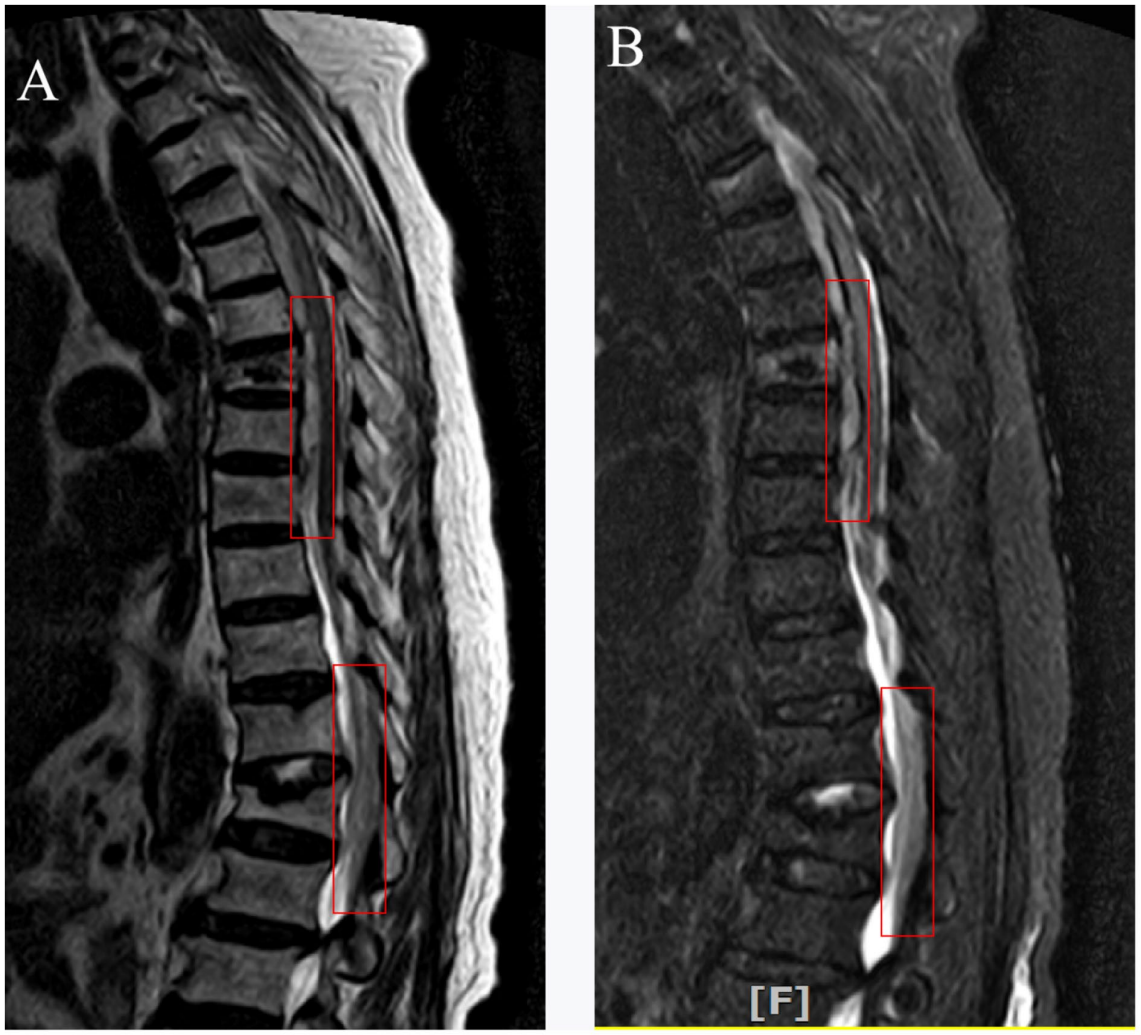
Two weeks later, thoracic MRI revealed less subdural hematoma in front of the spinal cord at the T4-8 vertebral plane and less epidural hematoma in the back of the spinal cord at the T12-L2 vertebral plane (Red box marked).

The present case describes a 90-year-old female patient presenting with prominent thoracic and back pain, intact sensory and motor functions in the lower limbs, as well as grade 5 muscle strength bilaterally in the lower extremities. Preoperative MRI revealed compression fractures in the 7th thoracic vertebrae, indicating the need for surgical intervention. The patient had a history of hypertension and was on long-term oral administration of amlodipine besylate for blood pressure control, which resulted in stable blood pressure management. Preoperative blood routine and coagulation function were normal. The patient experienced blood pressure fluctuations during kyphoplasty, up to 170/190–90/99 mmHg. One hour after the operation, the patient developed severe pain in the chest and back with loss of sensation below the xiphoid process (ASIA grade A), which lasted for 10 min before complete recovery (ASIA grade E). Emergency thoracolumbar MRI revealed subdural and extradural hemorrhage (Figure 1). Emergency decompression surgery was planned to save the patient’s neurological function, but the patient’s family refused the operation. About 1 day later, the blood pressure increased again, about 175–180/85-95 mmHg, and the patient again developed motor and sensory disturbances (ASIA grade A). About 20 h later, the sensation in both legs returned to normal. A repeat thoracolumbar MRI 2 weeks later showed that the hemorrhage in the spinal canal had been significantly absorbed (Figure 2). The blood pressure reduction was achieved using amlodipine besylate, nerve nutrition was provided through mecobalamin supplementation, pain relief was managed with non-steroidal drugs, and other symptomatic treatments were administered. At discharge, the patient’s neurological function partially recovered, with grade V muscle strength of the right lower limb and grade III muscle strength of the left lower limb, and the urination and defecation returned to normal (ASIA grade D).

The left approach was used for surgery, and preoperative C-arm fluoroscopy positioning was used. The C-arm X-ray machine was used to scan the surgical segments, and the scanning image data were transmitted to the orthopedic surgical robot workstation. After local anesthesia, a guide pin was drilled into the left pedicle of the seventh thoracic vertebra with the assistance of the robot. The balloon was inserted to expand the vertebral body, and the height of the vertebral body was partially restored under C-arm fluoroscopy. Bone cement (2.5 mL) was pushed into the vertebral body (see Table 1).

**Table 1:** Timeline showing the events and the neurological findings.

Time	The neurological findings
Pre-operation	Normal movement and sensation in the lower extremities
1 h after PKP	No sensation below xiphoid process, complete loss of Lower limb movement
70 min after PKP	Normal movement and sensation in the lower extremities
24 h after PKP	Complete loss of sensation and movement in the lower extremities
Status on discharge	Numbness in both lower limbs, muscle strength in the right lower limb was grade V, muscle strength in the left lower limb was grade III, and urine and bowels returned to normal

## Discussion and conclusions

Spinal cord injury is the most serious complication after PKP because it can lead to severe neurological dysfunction (9). It affects the quality of life of patients and even endangers their lives. Currently, relevant literature has reported that puncture errors directly lead to spinal cord injury, and bone cement leakage compressions the spinal cord resulting in spinal cord injury (10). Acute hematoma compression of the spinal cord due to coagulation dysfunction has been reported in the literature (11, 12) (see Table 2).

**Table 2:** Cases of spinal subdural hematoma after PKP reported in literature.

Case	Age, gender	Fracture level	Cause analysis	Clinical outcome
Tropeano et al. (1)	63 yr., male	L1, L3	Not described	Recovery after surgical decompression
Zou et al (11)	64 yr., female	L1	Anticoagulation	Recovery after surgical decompression
von der Brelie et al. (12)	63 yr., male	T12	Anticoagulation	Recovery after surgical decompression
Curković et al. (10)	49 yr., female	T8	venous congestion	Recovery after surgical decompression
Fang et al. (7)	69-84 yr. 5 female,1 male	T8-12	The piercing damage	Recovery after surgical decompression

**Figure 3:**
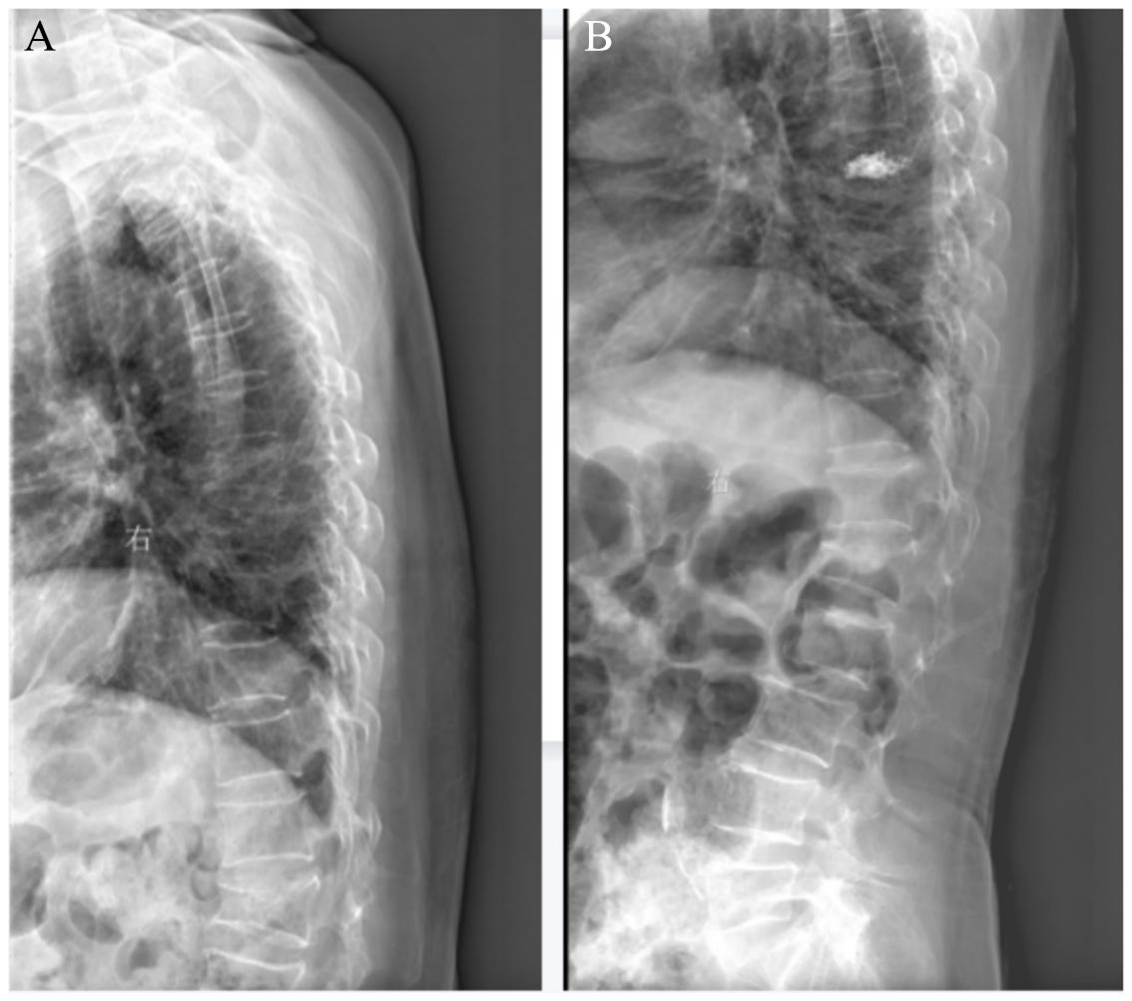
**(A)** Preoperative X-ray suggested the 7th thoracic vertebrae compression fracture. **(B)** After PKP of the 7th thoracic vertebra.

**Figure 4:**
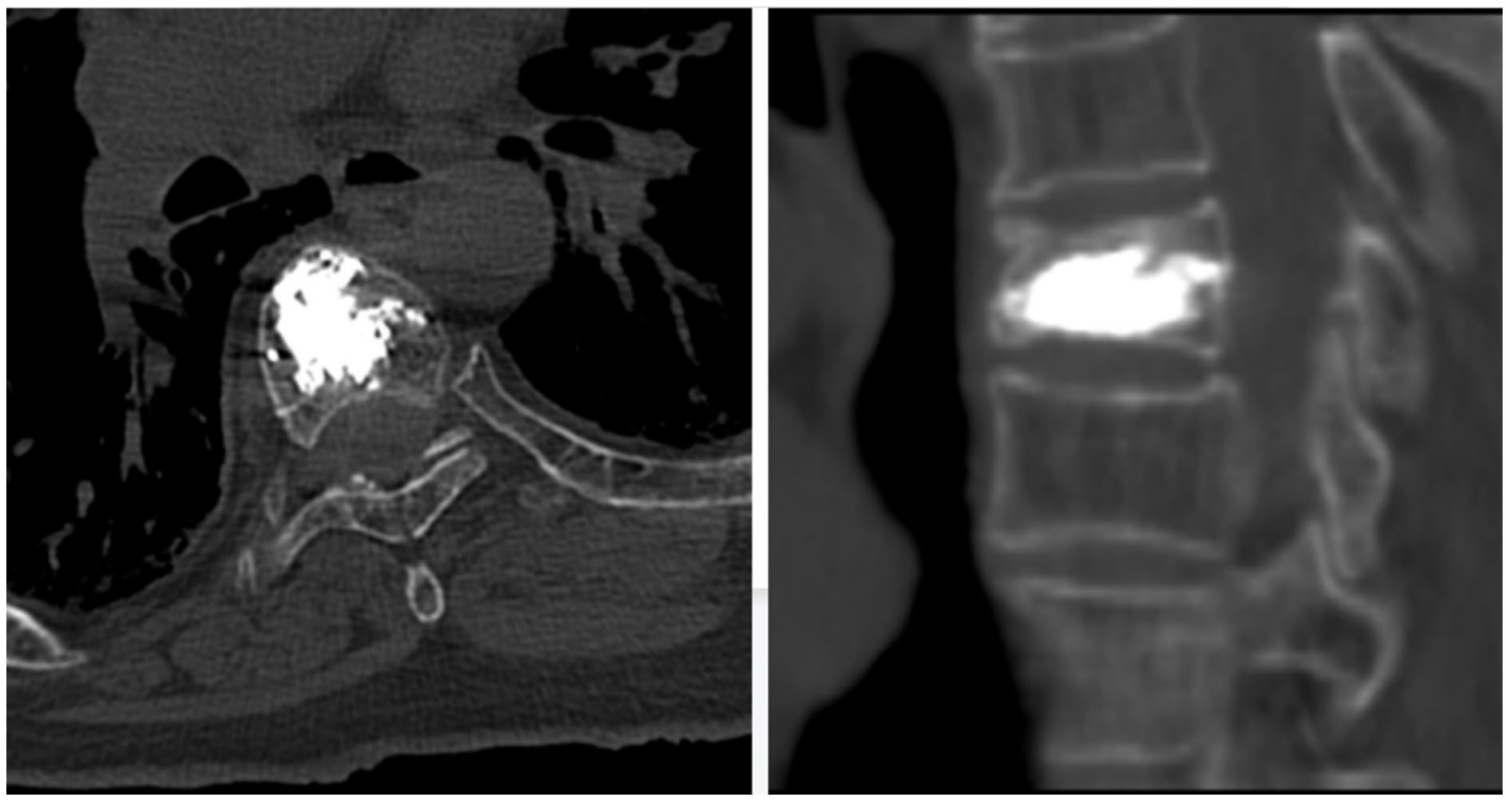
Postoperative CT scan showed no cement leakage into the spinal canal in sagittal and transverse views.

The patient we provided did not have sensory and motor disorders during the operation, and an intraoperative puncture did not find a puncture needle protruding into the spinal canal. Intraoperative computed tomography (CT) can help us find out whether there is a leakage of bone cement. Secondly, postoperative fluoroscopy and CT (Figures 3, 4) do not find puncture into the spinal canal or leakage of bone cement (2). The amount of bone cement we injected was also much less than that reported in the literature. The patient’s preoperative coagulation function was normal, and the patient had no coagulation dysfunction or long-term oral antiplatelet drugs. By analyzing the postoperative MRI of the patient, we found that the subdural/epidural hematoma of the patient presented long segmental distribution and was not in the same plane. We traced the patient’s pre-onset and post-onset medical history, and the patient showed large fluctuations in blood pressure during the operation. Unbearable back pain, decreased muscle strength in the lower extremities, and sensory impairments begin about an hour after surgery. After the corresponding conservative treatment, the patient’s muscle strength and sensation improved. A day later, the neurological dysfunction returned, and blood pressure fluctuated before the onset of the disease. Therefore, we believe that the most likely cause of this patient’s hemorrhage is the rupture of spinal cord blood vessels caused by large fluctuations in blood pressure.

By reviewing the literature, Kao et al. (13) reported that preoperative hypertension is a high-risk factor for epidural hematoma after spinal surgery. Yasushi et al. (14) also showed that preoperative hypertension was a high-risk factor for postoperative epidural hematoma by studying the occurrence of epidural hematoma after lumbar decompression surgery. Yamada et al. (15) showed that blood pressure fluctuation of more than 50 mmHg during anesthesia extubation was a high-risk factor for postoperative epidural hematoma. Tropeano et al. (1) suggested that spinal venous plexus congestion may be a possible factor leading to subdural hematoma after surgery. Previous studies have shown that actions such as coughing that cause sudden increases in chest and abdominal pressure may lead to rupture of spinal vein congestion (16, 17). With the increase of age, the quality of blood vessels also decreases, and pain, surgery, and other irritants are more likely to induce blood pressure fluctuations in this case, the patient had an increase in blood pressure before two neurological disorders. In combination with the pathophysiological mechanisms reported in the current literature, we conclude that a short and large fluctuation of blood pressure leads to an instantaneous increase in the pressure of the spinal cord venous plexus, resulting in blood vessel congestion, rupture, and bleeding, but the specific pathogenesis is still unclear.

Fang et al. (7) performed decompression surgery on 6 patients with spinal cord injury after percutaneous kyphoplasty, and the neurological function recovered well after surgery. The authors believe that timely surgery is more conducive to the recovery of spinal cord function. Baek et al. (8) believe that decompression surgery should be performed in time for patients with sensory and motor dysfunction unless there is coagulation dysfunction or functional improvement. Sirin et al. (18) provided a case of a patient with postoperative epidural hematoma, whose neurological dysfunction gradually recovered within 12 h. So the patient had a complete neurological recovery after 25 days of conservative treatment. However, the authors concluded that the continued deterioration of nerve function still required decompression surgery. The cases we provide for the disease are due to poor blood pressure control and advanced age, and the family refused decompression surgery. In particular, the second time the patient has a complete spinal cord injury, our choice of treatment is the same. Fortunately, after pain relief, control of blood pressure, nutrition, and other drugs, the patient’s neurological function is significantly better than before.

Therefore, we believe that perioperative blood pressure should be well controlled. In particular, to avoid sharp fluctuations in blood pressure caused by pain and other reasons during the operation. To avoid acute intraspinal bleeding caused by intraspinal vascular rupture after operation, which may lead to serious neurological dysfunction.

## Data Availability

The original contributions presented in the study are included in the article/supplementary material, further inquiries can be directed to the corresponding author.

## References

[1] Tropeano MP La Pira B Pescatori L Piccirilli M. Vertebroplasty and delayed subdural cauda equina hematoma: review of literature and case report. World J Clin Cases. (2017) 5:333–9. doi: 10.12998/wjcc.v5.i8.333, PMID: 28868305 PMC5561502

[2] Cosar M Sasani M Oktenoglu T Kaner T Ercelen O Kose KC. The major complications of transpedicular vertebroplasty. J Neurosurg Spine. (2009) 11:607–13. doi: 10.3171/2009.4.SPINE08466, PMID: 19929366

[3] Fourney DR Schomer DF Nader R Chlan-Fourney J Suki D Ahrar K. Percutaneous vertebroplasty and kyphoplasty for painful vertebral body fractures in cancer patients. J Neurosurg. (2003) 98:21–30. doi: 10.3171/spi.2003.98.1.0021, PMID: 12546384

[4] Lee KD Sim HB Lyo IU Kwon SC Park JB. Delayed onset of spinal subdural hematoma after vertebroplasty for compression fracture: a case report. Korean J Spine. (2012) 9:285–8. doi: 10.14245/kjs.2012.9.3.285, PMID: 25983834 PMC4431021

[5] Zhong WY Chen HF You C Li J Liu Y Huang SQ. Spontaneous spinal epidural hematoma. J Clin Neurosci. (2011) 18:1490–4. doi: 10.1016/j.jocn.2011.02.039, PMID: 21920757

[6] Vats HS McKiernan FE. Infected vertebroplasty: case report and review of literature. Spine. (2006) 31:E859–62. doi: 10.1097/01.brs.0000240665.56414.88, PMID: 17047535

[7] Fang M Zhou J Yang D He Y Xu Y Liu X. Management and outcomes of spinal epidural hematoma during vertebroplasty: case series. Medicine. (2018) 97:e10732. doi: 10.1097/MD.0000000000010732, PMID: 29794750 PMC6393141

[8] Baek BS Hur JW Kwon KY Lee HK. Spontaneous spinal epidural hematoma. J Korean Neurosurg Soc. (2008) 44:40–2. doi: 10.3340/jkns.2008.44.1.40, PMID: 19096655 PMC2588288

[9] Xu Z Hao D Liu T He B Guo H He L. Cause analysis of open surgery used after percutaneous Vertebroplasty and Kyphoplasty. Med Sci Monitor. (2016) 22:2595–601. doi: 10.12659/MSM.898463, PMID: 27444135 PMC4968613

[10] Curković B Grazio S Babić-Naglić D Anić B Vlak T Hanih M. Recommendations of the Croatian Society for Rheumatology for prevention, diagnostics and treatment of post-menopausal osteoporosis. Reumatizam. (2008) 55:26–30. PMID: 19024267

[11] Zou P Gong HL Wei JM Wei DM Qian LX Liu P. Spinal epidural hematoma after percutaneous Kyphoplasty: case report and literature review. J Pain Res. (2020) 13:2799–804. doi: 10.2147/JPR.S280650, PMID: 33173329 PMC7648559

[12] von der Brelie C Fiss I Rohde V. Multilevel spinal combined subdural/subarachnoid hemorrhage resulting in paraplegia: an unusual complication of Kyphoplasty. J Neurol Surg Part A. (2019) 80:220–2. doi: 10.1055/s-0038-1676594, PMID: 30708389

[13] Kao FC Tsai TT Chen LH Lai PL Fu TS Niu CC. Symptomatic epidural hematoma after lumbar decompression surgery. Eur Spine J. (2015) 24:348–57. doi: 10.1007/s00586-014-3297-8, PMID: 24760464

[14] Fujiwara Y Manabe H Izumi B Harada T Nakanishi K Tanaka N. The impact of hypertension on the occurrence of postoperative spinal epidural hematoma following single level microscopic posterior lumbar decompression surgery in a single institute. Eur Spine J. (2017) 26:2606–15. doi: 10.1007/s00586-017-5165-9, PMID: 28597302

[15] Yamada K Abe Y Satoh S Yanagibashi Y Hyakumachi T Masuda T. Large increase in blood pressure after Extubation and high body mass index elevate the risk of spinal epidural hematoma after spinal surgery. Spine. (2015) 40:1046–52. doi: 10.1097/BRS.0000000000000876, PMID: 25768686

[16] Ozdemir O Calisaneller T Yildirim E Caner H Altinors N. Acute spontaneous spinal subdural hematoma in a patient with bilateral incarcerated inguinal hernia. Joint Bone Spine. (2008) 75:345–7. doi: 10.1016/j.jbspin.2007.05.019, PMID: 18337142

[17] Kim JS Lee SH. Spontaneous spinal subarachnoid hemorrhage with spontaneous resolution. J Korean Neurosurg Soc. (2009) 45:253–5. doi: 10.3340/jkns.2009.45.4.253, PMID: 19444355 PMC2682125

[18] Sirin S Arslan E Yasar S Kahraman S. Is spontaneous spinal epidural hematoma in elderly patients an emergency surgical case? Turk Neurosurg. (2010) 20:557–60. doi: 10.5137/1019-5149.JTN.2338-09.1, PMID: 20963712

